# Silent Persistence: Molecular Evidence of Clonal Transmission in Fluconazole-Resistant *Candida parapsilosis* Hospital Outbreaks over Decades

**DOI:** 10.3390/jof11110802

**Published:** 2025-11-12

**Authors:** Cihan Semet, Esra Kazak, Seçil Ak-Aksoy, Harun Ağca, Beyza Ener

**Affiliations:** 1 Department of Infectious Diseases and Clinical Microbiology, Faculty of Medicine, Bursa Uludağ University, Bursa 16059, Turkey; semetcihan@gmail.com; 2 Department of Microbiology, Faculty of Medicine, Bursa Uludağ University, Bursa 16059, Turkey

**Keywords:** *Candida parapsilosis*, microsatellite genotyping, clonal spread

## Abstract

Fluconazole-resistant *Candida parapsilosis* has emerged as a significant nosocomial pathogen, contributing to extensive outbreaks with severe clinical implications. Despite increasing evidence of clonal transmission, the genetic mechanisms that facilitate the persistence of hospital reservoirs remain inadequately characterized. We aimed to characterise the long-term molecular epidemiology of fluconazole-resistant *Candida parapsilosis* bloodstream isolates (n = 47) collected between 1997 and 2019 at a tertiary centre. All isolates underwent microsatellite analysis using three polymorphic markers (CP1, CP4, B5). Genetic diversity, temporal distribution, and clonal relationships were assessed through phylogenetic analysis and discriminatory power calculations. Microsatellite analysis revealed minimal genetic diversity (combined discriminatory power: 0.7114), with only six distinct genotypes identified. Two dominant clones (Genotype-1: 23.4%, Genotype-2: 46.8%) persisted throughout the study, showing apparent spatiotemporal clustering in surgical and intensive care units. Phylogenetic analysis demonstrated tight genetic clustering, consistent with prolonged clonal persistence across multiple years and clinical departments. Our findings provide strong molecular evidence consistent with persistent, multi-year clonal transmission; however, definitive confirmation will require higher-resolution genomics and epidemiologic linkage. These results underscore the need to strengthen infection-control practices to curtail sustained clonal persistence within the hospital.

## 1. Introduction

*Candida albicans*, traditionally considered to be the most common species among the causative agents of candidemia, is increasingly being replaced by non-albicans Candida (NAC) species such as *C. glabrata, C. tropicalis* and *C. parapsilosis* [1,2]. The increase in NAC species is thought to be due to multiple factors. The widespread use of broad-spectrum antibiotics disrupts the normal microbiota and creates a favorable environment for the development of opportunistic fungal infections [3]. *C. parapsilosis*, which is common in the skin flora and can settle on foreign surfaces such as catheters, has started to be seen frequently among the healthcare-associated candidemia agents among NAC species with the effect of increasing invasive procedures [4]. Current guidance recommends initial echinocandin therapy for candidaemia, with step-down to fluconazole upon documented susceptibility, including infections due to *C. parapsilosis* [5]. However, the incidence of *C. parapsilosis* strains showing fluconazole resistance has increased significantly worldwide in recent years [6]. This resistance limits clinical treatment options and negatively affects the clinical prognosis of patients [7]. Fluconazole-resistant *C. parapsilosis* strains cause an increase in morbidity and mortality rates by decreasing treatment success, especially in intensive care units and patients with invasive medical devices [8]. Therefore, monitoring fluconazole resistance and preventing the spread of resistant strains in the hospital environment is of great importance.

*C. parapsilosis* has been associated with fungemia outbreaks in the hospital environment and has been frequently isolated from the skin flora of healthcare workers and surfaces in the hospital environment [9,10,11]. The fact that it has the capacity to form biofilms by colonizing on hospital surfaces and invasive medical devices significantly increases the risk of cross-infection by increasing the virulence of the pathogen [12]. Mechanistically, these biofilms are characterized by a mannan–glucan extracellular matrix (containing β-1,3-glucan) and Bcr1/Efg1-regulated adhesins (e.g., Rbt1-like proteins), which together promote surface persistence and device colonization [13].

In various studies, it has been found that fluconazole-resistant *C. parapsilosis* strains are genetically similar and this supports inter-patient transmission [14]. It has been reported that resistant strains isolated from the hands of hospital staff and environmental surfaces are genetically similar to strains isolated from patients [15,16]. In addition, genetic analyses have shown that certain resistant genotypes remain persistent in the hospital environment for years and are transmitted to different patients [17]. This situation has become particularly evident in intensive care units and neonatal units [14,18].

Microsatellite genotyping is a widely used method for assessing genetic relationships and inferring clonal structure among fluconazole-resistant *C. parapsilosis* strains, particularly in longitudinal cohorts. Analyses performed with this method show that resistant strains found in the hospital environment and on the hands of healthcare workers are genetically similar and indicate transmission routes [19]. In this study, we aimed to genotype fluconazole-resistant *C. parapsilosis* strains isolated from patients diagnosed with candidemia in our hospital over a 22-year period by microsatellite analysis method and to evaluate the genetic similarity between them.

## 2. Materials and Methods

Ethics committee approval was obtained from Bursa Uludag University, Non-Interventional Clinical Research Ethics Committee (Decision No: No. 2020-10/15). This study was conducted in a tertiary level reference hospital with intensive care units and clinical services. This was a single-centre cohort at Bursa Uludağ University Faculty of Medicine Hospital (Bursa, Türkiye). All 47 fluconazole-resistant *C. parapsilosis* bloodstream isolates (1997–2019) were obtained from candidemia episodes at this hospital, and all cultures were processed in the same clinical microbiology laboratory.

### 2.1. DNA Isolation and Polymerase Chain Reaction (PCR)

In our study, 47 blood isolates previously identified as *C. parapsilosis* sensu stricto, which were isolated from patients diagnosed with candidemia between 1997 and 2019 and found to be fluconazole-resistant, were used [20]. All isolates derived from candidemia episodes in the same tertiary-care hospital and were processed in the same clinical microbiology laboratory, confirming the single-centre nature of the cohort. DNA isolation from the isolates was performed using UltraClean Microbial DNA Isolation Kit (Qiagen, Valencia, CA, USA) according to the manufacturer’s recommendations. The concentration and purity of the DNA samples obtained were evaluated with a Beckman Coulter DU-640 spectrophotometer using absorbance measurement at 260/280 nm wavelengths.

### 2.2. Antifungal Susceptibility Test

*C. parapsilosis* species complex isolates grown on blood cultures (BACTEC-FX: Becton-Dickinson, Sparks, MD, USA) were identified using germ tube test, morphology and biochemical profile (API ID 32C; BioMérieux, Craponne, France) on corn flour tween 80 and chromogenic culture media. Antifungal susceptibility testing was performed using the microdilution method according to Clinical and Laboratory Standards Institute (CLSI) guidelines. against fluconazole, voriconazole, itraconazole, posaconazole, amphotericin B, and anidulafungin. All isolates were archived at −80 °C and re-cultivated prior to testing. Antifungal susceptibility testing followed CLSI broth microdilution standards, and interpretation used CLSI breakpoints (M27 in earlier years; most recent updates such as M27M44S/M60). According to the CLSIM27M44S/M60 document, strains with a minimum inhibitory concentration MIC ≥ 4 µg/mL were considered fluconazole-non susceptible (MIC = 4 µg/mL sensitive dose-dependent; MIC ≥ 8 µg/mL resistant) [21,22]. Although CLSI specifies 4 µg/mL as dose-dependent susceptible, studies have shown that resistance starts at this MIC value and resistance genes are present. Therefore, in this study, all isolates with ≥4 µg/mL were expressed as fluconazole non-susceptible [23,24].

### 2.3. Microsatellite Genotyping Analysis

For the microsatellite genotyping analysis, PCR was performed with primers of fluorescently labeled microsatellite markers (CP1, CP4, and B5) using the genomic DNA obtained. Primer sequences and PCR conditions followed Sabino et al. [25]; forward primers were fluorescently labeled. The exact sequences (5′→3′) for CP1, CP4, and B5 are provided in Supplementary Table S1. The reaction mixture, in a total volume of 10 μL, contained 50–100 ng genomic DNA, 1.25 units of Taq polymerase, 0.8 mM of each dNTP, 1.5 mM MgCl_2_, and 0.5 μM of each primer. Each PCR batch included a positive control (*Candida parapsilosis* ATCC 22019) and a no-template control processed alongside study isolates.

High-quality PCR products were processed using the “Sample Loading Solution” and “DNA Size Standard Kit” (Beckman Coulter, Fullerton, CA, USA), and fragment analysis was performed with the “CEQ 8000 Genetic Analysis System” (Beckman Coulter Inc., Fullerton, CA, USA). After analysis, the data of each sample were comparatively evaluated using CEQ 8000 software version 3.0, the integrated analysis software of the device.

### 2.4. Phylogenetic Tree Construction

The phylogenetic tree for microsatellite genotyping analysis was constructed using “BioNumerics” version 6.6 (Applied Maths, NV) software. Allele profiles were analysed in BioNumerics as categorical (multistate) characters; pairwise similarity was calculated with the categorical (matching) coefficient with default optimization (1.0%) and position tolerance (1.0%), and the dendrogram was constructed using UPGMA clustering. In the resulting UPGMA dendrogram, branch lengths represent categorical distance (1 − similarity), and the scale bar indicates percent similarity. Allele numbers, repeat numbers and allele frequencies were calculated with “CONVERT” software version 1.31. Hardy–Weinberg equilibrium and Chi-square test results were analyzed using “Genepop” software version 4.2. Discrimination power (DP) was calculated according to the formula proposed by Hunter et al. [26].

## 3. Results

Antifungal susceptibility profile of 47 *C. parapsilosis* strains from patients with candidemia is shown Table 1. Forty-seven fluconazole-resistant *C. parapsilosis* strains isolated from patients with candidemia were analyzed at the molecular level using CP1, CP4 and B5 microsatellite markers; the genetic diversity and possible epidemiological relationships of the isolates were comprehensively evaluated by microsatellite typing method.

The discrimination levels of three microsatellite markers differed. The CP1 marker revealed three different alleles ranging from 224 to 302 base pairs; the number of repeats ranged from 1 to 40 and allele frequencies ranged from 0.0213 to 0.9574. The DP of this marker was calculated as 0.0842; although heterozygosity was not observed, it provided basic information on genetic variation. The CP4 marker showed a higher level of diversity; four alleles ranging between 249 and 286 base pairs were detected, the number of repeats was between 1 and 19, and the allele frequencies were between 0.0213 and 0.8511. In CP4 marker, 4.25% heterozygosity was observed, and the DP was determined as 0.2683. The B5 marker stood out as the marker with the highest discrimination power (DP: 0.5495); three alleles ranging between 140 and 148 base pairs were identified, repeat numbers ranged between 1 and 5, and allele frequencies ranged between 0.1277 and 0.6170. Heterozygosity was not detected in this marker. When all markers were considered, the total DP was calculated as 0.7114 (Table 2).

**Figure 1:**
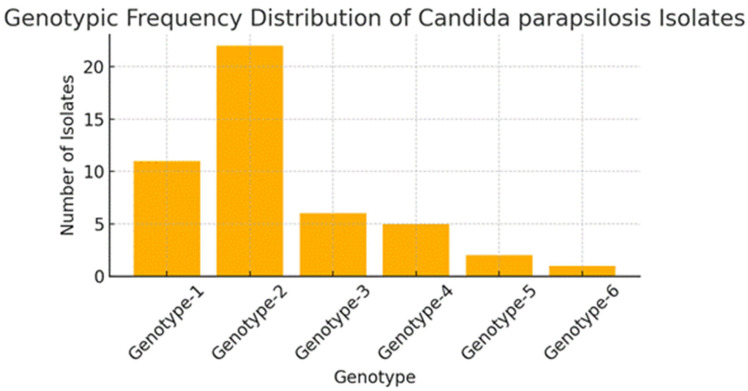
Genotype frequency distribution of *Candida parapsilosis* isolates.

A total of 47 fluconazole-resistant *C. parapsilosis* isolates were genotyped and six different genotypes were identified. When the genotype distribution was analyzed, it was found that the most common genotype was Genotype-2 and it was the most represented group with a total of 22 isolates (46.8%). Genotype-2 was followed by Genotype-1 with 11 isolates (23.4%). The distribution of other genotypes was as follows: Genotype-3 (6 isolates), Genotype-4 (4 isolates), Genotype-5 and Genotype-6 (2 isolates each). The average number of isolates per genotype was 12.7% (Figure 1) (Supplementary Table S1). Fluconazole MICs for all 47 isolates, stratified by genotype, are provided in Supplementary Figure S1.

**Figure 2:**
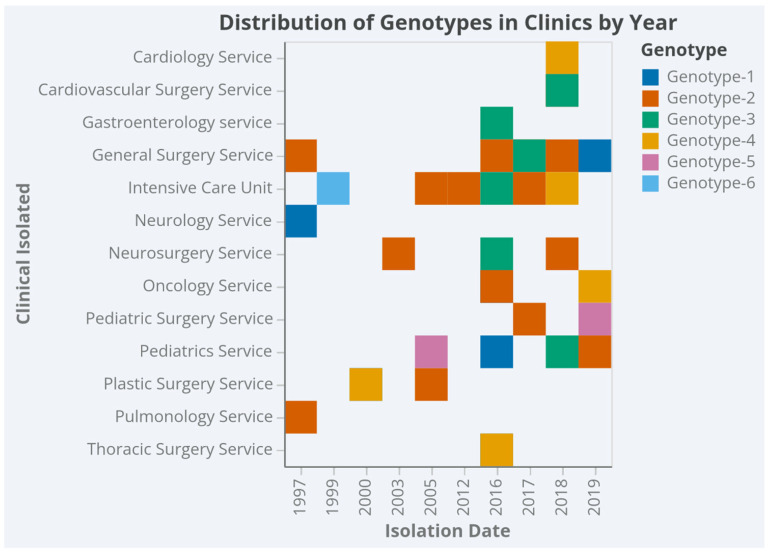
Genotype (G1–G6) distribution by clinical service and isolation year.

The distribution of genotypes in clinics and years is shown in Figure 2. Accordingly, Genotype-2 was the most frequently isolated type in General Surgery and Intensive Care Unit in 2017 and 2018, while Genotype-1 was the predominant type in internal clinics, especially in Oncology and Neurology, in the 2000–2016 period. Genotypes-3, -4, -5 and -6 were rarely isolated in different clinical services and in variable years (Figure 2) (Supplementary Table S2, Supplementary Figure S2).

**Figure 3:**
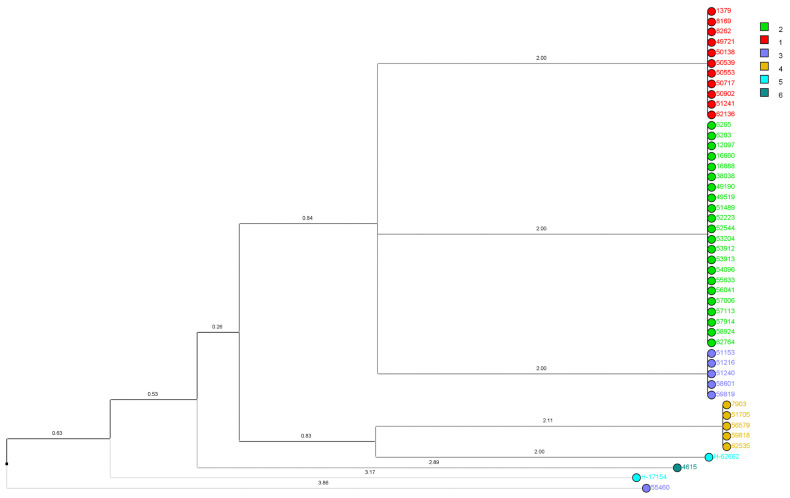
UPGMA dendrogram of multilocus microsatellite genotypes. Similarity coefficient: categorical (matching); optimization 1.0%, position tolerance 1.0% (BioNumerics v6.6). Branch lengths represent categorical distance (1 − similarity) computed from CP1, CP4, and B5 allele profiles; scale bar indicates percent similarity.

**Figure 4:**
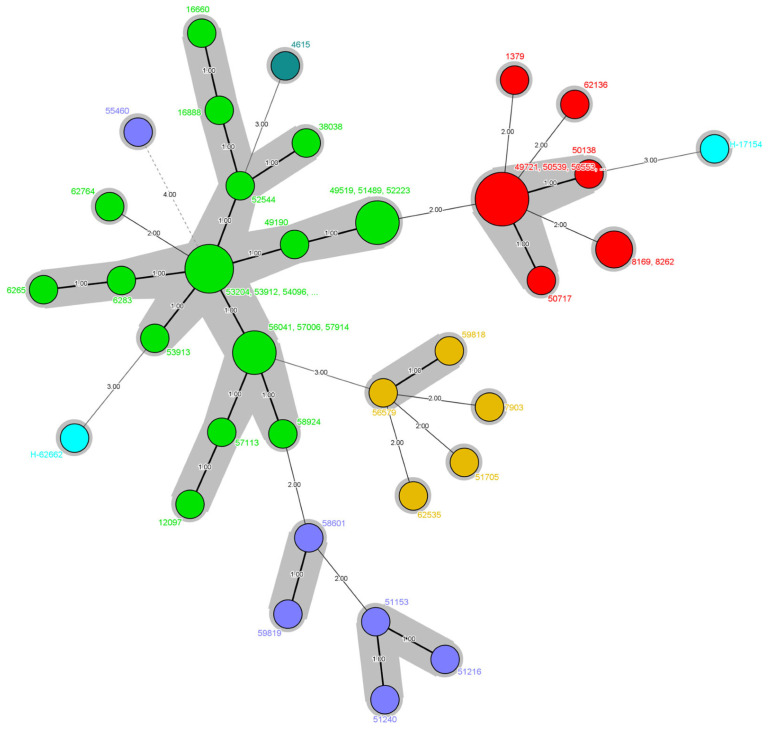
Phylogenetic tree showing genetic similarities and relationships among isolates, with genotypes represented by different colors and closely related isolates shaded and highlighted in gray. Grey shading indicates a pairwise allelic difference ≤1 across CP1/CP4/B5 (single-locus step).

Phylogenetic relationships and genetic similarities among isolates were visualized through a dendrogram (Figure 3) and phylogenetic tree (Figure 4). Dendrogram analysis revealed clear clustering patterns, with Genotype-2 in particular exhibiting a highly compact and homogeneous structure. In contrast, Genotype-1 showed a more disorganized genetic distribution, while Genotypes-3, -4, -5 and -6, which were observed less frequently, showed more genetic heterogeneity. Phylogenetic tree analysis also supported these findings; genotypes were identified with different colors and genetically close isolates were highlighted with gray shading with a genetic distance of ≤1.

## 4. Discussion

In this study, we genotyped 47 fluconazole-resistant isolates of *C. parapsilosis*, a pathogen recently designated as “high priority” by the World Health Organization (WHO) due to its significant public health threat [27], using the CP1, CP4, and B5 markers and observed a limited diversity among them, and the three-locus panel demonstrated moderate discriminatory power (DP = 0.7114). The CP1 marker was nearly monomorphic, exhibiting a DP of 0.0842, indicating 100% homozygosity. The CP4 marker showed moderate polymorphism, with a DP of 0.2683 and 4.25% heterozygosity. Although the B5 marker demonstrated the highest DP at 0.5495, it also displayed complete homozygosity. The combined DP of 0.7114 is notably lower than the approximately 0.99 reported for four-locus panels [25].

In our comparison of data with international multilocus schemes, we noted significantly lower DP. Sabino et al. utilized a four-locus panel (CP1, CP4, CP6, and B5) on 236 *C. parapsilosis* isolates, reporting DP values of approximately 0.85 for CP1, 0.90 for CP4, and 0.86 for B5, resulting in a combined DP of 0.99 and 192 distinct genotypes. In contrast, our findings revealed a DP of only 0.084 for CP1, 0.2683 for CP4, and 0.55 for B5—all loci being homozygous—culminating in a combined DP of 0.7114 [25]. Similarly, Desnos-Ollivier et al. identified around 30 multilocus genotypes and reported a panel DP close to 0.97 in isolates from France and Uruguay [28]. These elevated DP values suggest a significant degree of allelic diversity. In contrast, the lower metrics observed in our study imply that many of our fluconazole-resistant isolates possess identical or closely related genotypes, indicative of a predominantly clonal population. These results emphasize that three loci may not be adequate to capture the full genetic heterogeneity of persistent *C. parapsilosis* strains in our hospital environment. As a pragmatic next step, a four-locus scheme incorporating CP6 or CP10 in combination with CP1/CP4/B5 may increase discriminatory power and reduce genotype conflation; where available, MLST or whole-genome sequencing can further refine relatedness estimates.

With a DP of 0.7114, our three-locus panel performed below the levels typically reported for four-locus microsatellite schemes. We attribute this to several non–mutually exclusive factors. First, a three-locus panel is inherently less resolving than a four-locus assay. Second, a single multilocus genotype predominated across multiple years and clinical wards, consistent with clonal dominance and the consequent reduction in allelic diversity. Third, by focusing on archived fluconazole-resistant bloodstream isolates, we likely introduced selection toward azole-exposed strains and may have under-ascertained other contemporaneous genotypes. Finally, the lack of environmental and healthcare-worker sampling may have missed additional reservoirs and diversity.

Previous research has shown that fluconazole-resistant *C. parapsilosis* clones may emerge and, under selective pressures, become predominant in clinical settings [29]. Some of these resistant strains can persist for several years; for instance, a study from Canada documented a single resistant strain that circulated for 5.5 years, causing ongoing infections [30]. This clonal expansion significantly contributes to the low diversity observed in our collection. Previous genomic studies have highlighted a predominantly clonal population structure, as indicated by significant deviations from the Hardy–Weinberg equilibrium and heterozygosity levels 25 to 70 times lower than those found in other diploid Candida species [26,31]. Consequently, slight genetic variation accumulates during replication, allowing identical clones to infect multiple patients. Our cohort’s lack of variation at loci CP1 and B5, alongside only minimal polymorphism at CP4, suggests that our 47 isolates originate from several ancestral clones, with CP1 as a “clonal trace”. While three-locus microsatellite panels are commonly used and deemed sufficiently discriminatory, our findings indicate they may underestimate the true genetic diversity of long-standing resistant populations [28,32].

We note that *C. parapsilosis* easily colonizes hospital environments through the hands of healthcare workers and invasive medical devices, resulting in infection clusters dominated by specific clones [33]. Intensive care units for neonates and adults are especially susceptible to such outbreaks [34,35,36]. For instance, a Turkish hospital reported an outbreak involving a single genotype, while a Chinese study encompassing ten hospitals identified 122 different genotypes, with 32 of those forming clonal clusters—one specific MT42 clone was responsible for infections in 22 neonates within a single NICU [33,37]. These findings highlight how swiftly a single clone can spread under favorable conditions. Additionally, the high genotypic similarity observed among our 47 isolates is consistent with nosocomial clonal persistence; however, direct epidemiological confirmation is still pending.

As a diploid organism, *C. parapsilosis* has the potential to carry two different alleles per locus, which would reflect sexual recombination or admixture [38]. However, its predominantly clonal, asexual lifecycle results in very low rates of heterozygous SNPs and microsatellite loci [31]. In our collection, CP1 and B5 were completely homozygous (0% heterozygosity), while CP4 exhibited only a single heterozygous isolate (4.25%). This evidence is consistent with these 47 isolates originating from a largely monotypic, clonal population; however, it does not by itself conclusively demonstrate direct transmission chains.

In contrast to the 5–7% average genotype representation reported by Sabino et al. [25], our isolates exhibited an average of 12.7%, consistent with a predominantly clonal population and a panel showing moderate discriminatory power (DP = 0.7114). Genotype 2 (n = 22) accounted for 46.8% of our cohort, suggesting significant clonal expansion. Such outbreaks predominantly involving a single genotype are well-documented in intensive and neonatal care settings [34]. The pronounced presence of Genotype 2 in our hospital implies dissemination from a common source or transmission chain, highlighting the urgent need to strengthen infection control measures, especially hand hygiene, catheter care, and protocols for invasive procedures [38].

Fluconazole-resistant *C. parapsilosis* infections are primarily found in high-risk environments—specifically, adult and neonatal ICUs, transplant units, and COVID-19 wards—where vulnerable patients are concentrated [39]. In our study, Genotype 2 was particularly prevalent in general surgery and intensive care unit during 2017–2018, aligning with findings that indicate how ICUs and surgical services—through invasive interventions such as central venous catheters and parenteral nutrition—facilitate clonal dissemination [40,41,42]. Resistant strains also formed clusters in the Chest Diseases and Pediatric Surgery units during the Ege University candidemia outbreak of 2019–2020 [43]. In contrast, Genotype 1 (n = 11) was detected in oncology and neurology wards between 2000 and 2016—areas characterized by immunosuppression, prolonged stays, and invasive procedures—underscoring distinct epidemiological patterns specific to different units [37,44]. The occasional detection of genotypes 3–6, which are typically associated with isolated or exogenous introductions, further suggests multiple entry points and heterogeneous transmission routes [19,33].

Our dendrogram and phylogenetic analyses revealed that Genotype 2 isolates form a compact, homogeneous cluster, consistent with nosocomial clonal persistence; nevertheless, conclusive demonstrations would require complementary epidemiologic linkage and higher-resolution typing. Similar clustering patterns have been observed in outbreaks of *C. parapsilosis* and are recognized as key indicators of in-hospital transmission, particularly in ICU and surgical ward environments [45,46]. In the phylogenetic tree, distinct genotypes were color-coded, and clusters with genetic distances of ≤1 were highlighted, further confirming the close relatedness of the dominant clones.

Several modifiable factors may facilitate long-term persistence of a dominant *C. parapsilosis* clone in hospital settings. First, hand carriage by healthcare workers and imperfect hand-hygiene adherence can sustain transmission for a species that readily colonises skin and abiotic surfaces [19]. Second, device management, especially central-line insertion and maintenance, hub disinfection, and the preparation and administration of parenteral nutrition, creates opportunities for biofilm-mediated survival and ward-level spread [47]. Third, antifungal prescribing patterns may impose selective pressure (e.g., sustained fluconazole exposure favouring fluconazole-non-susceptible lineages), while the intrinsically higher echinocandin MICs of *C. parapsilosis* can complicate empirical choices [48]. Finally, structural pressures such as high device-days, crowding, or staff turnover may lower the threshold for clone persistence [31]. Although we did not collect quantitative data on hand hygiene, device-care bundle compliance, or ward-level antifungal consumption, these mechanisms provide a realistic explanatory framework and suggest testable quality-improvement targets for future work.

We recognise several limitations. First, the retrospective, single-centre design and focus on fluconazole-non-susceptible bloodstream isolates may limit generalisability and introduce selection towards azole-exposed lineages. Second, the use of only three microsatellite loci (CP1, CP4 and B5) likely reduced allelic resolution relative to four-locus schemes. Third, because analyses relied on archival isolates, DNA degradation remains a potential source of artefact; although we did not directly quantify DNA integrity, isolates were re-cultivated prior to testing, each PCR batch included positive and no-template controls, and ambiguous or low-quality electropherograms were repeated, so degradation was not demonstrated but cannot be excluded and may have influenced allelic calls in a minority of samples. Fourth, we lacked environmental and healthcare-worker isolates and did not have patient-level spatiotemporal linkage (e.g., room/bed movement, device sharing). Fifth, we did not systematically assess MICs for other azoles or echinocandins across the archive, precluding analysis of cross-resistance patterns. Sixth, we did not perform targeted ERG11 sequencing or evaluate additional azole-resistance determinants (e.g., efflux regulation), preventing genotype–mechanism correlations. Consequently, while our three-locus panel (DP = 0.7114) provides strong molecular evidence of clonal persistence, it cannot by itself confirm direct transmission without complementary genomic (e.g., MLST/WGS) and epidemiologic corroboration.

In conclusion our data reveals limited genetic diversity among fluconazole-resistant *C. parapsilosis* isolates, with Genotype 2 and Genotype 1 dominating throughout the hospital over two decades—findings consistent with nosocomial clonal persistence, though not a definitive demonstration of direct transmission. These results emphasise the need for enhanced infection control measures, particularly in surgical and intensive care units. We advocate for future multicenter studies incorporating larger collections of isolates, expanded multilocus panels, and comprehensive patient clinical metadata to enhance DP and better elucidate the transmission dynamics of resistant *C. parapsilosis.* Future efforts to fully elucidate transmission dynamics should comprise multicentre, prospective collaborations that integrate high-resolution genomics (cgMLST/WGS), targeted ERG11 sequencing and standardized antifungal MIC panels with environmental and healthcare-worker sampling and harmonised process/stewardship metrics, thereby resolving transmission pathways and genotype-specific resistance mechanisms.

## Figures and Tables

**Table 1:** In vitro antifungal profile of 47 fluconazole resistant *Candida parapsilosis* strains.

Antifungal Agents	MIC Ranges (µg/mL)	MIC 50 (µg/mL)	MIC 90 (µg/mL)	Percent of Non-Susceptible /Non-Wild Type Isolates
Fluconazole	4–>64	8	32	100 (≥4 µg/mL)
Voriconazole	0.06–1	0.125	0.25	14.9 (≥0.25 µg/mL)
İtraconazole	0.06–0.5	0.06	0.25	none
Posaconazole	≤0.03 –0.125	0.06	0.125	none
Amphotericin B	0.5–1	0.5	1	none
Anidulafungin	0.5–2	0.5	1	none

**Table 2:** Characteristics of Microsatellite Markers.

Marker	Number of Alleles	Allele Sizes	Number of Repeats	Allele Frequencies	Number of Genotypes	Genotype Frequencies	Heterozygosity Rate (%)	DP *
CP1	3	224–302	1–40	0.0213–0.9574	3	0.0110–0.8123	0	0.0842
CP4	4	249–286	1–19	0.0213–0.8511	3	0.0222–0.6691	0.0425	0.2683
B5	3	140–148	1–5	0.1277–0.6170	3	0.0676–0.3901	0	0.5495
Total								0.7114

* DP: discriminatory power.

## Data Availability

The original contributions presented in this study are included in the article. Further inquiries can be directed to the corresponding author.

## Supplementary Materials

The following supporting information can be downloaded at: https://www.mdpi.com/article/10.3390/jof11110802/s1, Figure S1: MIC Values by Isolate ID and Genotype; Figure S2: Temporal distribution of genotypes by year; Table S1. Microsatellite allele lengths and genotype assignment; Table S2. Year and clinical service for each isolate with genotype.
