# Supplementary material for: Silent Persistence: Molecular Evidence of Clonal Transmission in Fluconazole-Resistant Candida parapsilosis Hospital Outbreaks over Decades
Source: J Fungi (Basel). 2025 Nov 12;11(11):802. doi: 10.3390/jof11110802 (PMC12653143; doi:10.3390/jof11110802)
Supplement: Supplementary file 1 [file jof-11-00802-s001.zip › Supplementary Table S2.pdf]

Table S2. Year and clinical service for each isolate with genotype

Year	Clinical service	Genotype
1997	Neurology Service	Genotype-1
1997	Pulmonology Service	Genotype-2
1997	General Surgery Service	Genotype-2
1999	Intensive Care Unit	Genotype-6
2000	Plastic Surgery Service	Genotype-4
2000	Plastic Surgery Service	Genotype-1
2000	Plastic Surgery Service	Genotype-1
2003	Neurosurgery Service	Genotype-2
2005	Plastic Surgery Service	Genotype-2
2005	Intensive Care Unit	Genotype-2
2005	Pediatrics Service	Genotype-5
2012	Intensive Care Unit	Genotype-2
2016	General Surgery Service	Genotype-2

2016	Oncology Service	Genotype-2
2016	Oncology Service	Genotype-1
2016	Pediatrics Service	Genotype-1
2016	Oncology Service	Genotype-1
2016	Oncology Service	Genotype-1
2016	Thoracic Surgery Service	Genotype-1
2016	Oncology Service	Genotype-1
2016	Gastroenterology Service	Genotype-3
2016	Neurosurgery Service	Genotype-3
2016	Intensive Care Unit	Genotype-3
2016	Oncology Service	Genotype-1
2016	Oncology Service	Genotype-2
2016	Thoracic Surgery Service	Genotype-4
2016	Oncology Service	Genotype-2

2017	Intensive Care Unit	Genotype-2
2017	General Surgery Service	Genotype-2
2017	General Surgery Service	Genotype-2
2017	Pediatric Surgery Service	Genotype-2
2017	General Surgery Service	Genotype-2
2017	General Surgery Service	Genotype-3
2017	General Surgery Service	Genotype-2
2018	General Surgery Service	Genotype-2
2018	Intensive Care Unit	Genotype-4
2018	General Surgery Service	Genotype-2
2018	Neurosurgery Service	Genotype-2
2018	General Surgery Service	Genotype-2
2018	Cardiovascular Surgery Service	Genotype-3
2018	Cardiovascular Surgery Service	Genotype-2

2018	Cardiology Service	Genotype-4
2018	Pediatrics Service	Genotype-3
2019	General Surgery Service	Genotype-1
2019	Oncology Service	Genotype-4
2019	Pediatric Surgery Service	Genotype-5
2019	Pediatrics Service	Genotype-2
