# Supplementary material for: Silent Persistence: Molecular Evidence of Clonal Transmission in Fluconazole-Resistant Candida parapsilosis Hospital Outbreaks over Decades
Source: J Fungi (Basel). 2025 Nov 12;11(11):802. doi: 10.3390/jof11110802 (PMC12653143; doi:10.3390/jof11110802)
Supplement: Supplementary file 1 [file jof-11-00802-s001.zip › Supplementary Table S1.pdf]

Table S1. Microsatellite allele lengths and genotype assignment

Isolate ID	CP1 A	CP1 B	CP4 A	CP4 B	B5 A	B5 B	Genotype
1379	240	240	259	259	148	148	Genotype-1
4615	302	302	259	259	145	145	Genotype-6
6265	240	240	259	259	145	145	Genotype-2
6283	240	240	259	259	145	145	Genotype-2
7903	240	240	277	277	145	145	Genotype-4
8169	240	240	259	259	148	148	Genotype-1
8262	240	240	259	259	148	148	Genotype-1
12097	240	240	259	259	145	145	Genotype-2
16660	240	240	259	259	145	145	Genotype-2
16888	240	240	259	259	145	145	Genotype-2
17154	240	240	249	286	148	148	Genotype-5
38038	240	240	259	259	145	145	Genotype-2
49190	240	240	259	259	145	145	Genotype-2

49519	240	240	259	259	145	145	Genotype-2
49721	240	240	259	259	148	148	Genotype-1
50138	240	240	259	259	148	148	Genotype-1
50539	240	240	259	259	148	148	Genotype-1
50553	240	240	259	259	148	148	Genotype-1
50717	240	240	259	259	148	148	Genotype-1
50902	240	240	259	259	148	148	Genotype-1
51153	240	240	259	259	140	140	Genotype-3
51216	240	240	259	259	140	140	Genotype-3
51240	240	240	259	259	140	140	Genotype-3
51241	240	240	259	259	148	148	Genotype-1
51489	240	240	259	259	145	145	Genotype-2
51705	240	240	277	277	145	145	Genotype-4
52223	240	240	259	259	145	145	Genotype-2

52544	240	240	259	259	145	145	Genotype-2
53204	240	240	259	259	145	145	Genotype-2
53912	240	240	259	259	145	145	Genotype-2
53913	240	240	259	259	145	145	Genotype-2
54096	240	240	259	259	145	145	Genotype-2
55460	224	224	259	259	140	140	Genotype-3
55633	240	240	259	259	145	145	Genotype-2
56041	240	240	259	259	145	145	Genotype-2
56579	240	240	277	277	145	145	Genotype-4
57006	240	240	259	259	145	145	Genotype-2
57113	240	240	259	259	145	145	Genotype-2
57914	240	240	259	259	145	145	Genotype-2
58601	240	240	259	259	140	140	Genotype-3
58924	240	240	259	259	145	145	Genotype-2

59818	240	240	277	277	145	145	Genotype-4
59819	240	240	259	259	140	140	Genotype-3
62136	240	240	259	259	148	148	Genotype-1
62535	240	240	277	277	145	145	Genotype-4
62662	240	240	249	286	145	145	Genotype-5
62764	240	240	259	259	145	145	Genotype-2
**Summary (genotype totals):** Genotype-1 **n = 11 (23.4%)**, Genotype-2 **n = 22 (46.8%)**, Genotype-3 **n = 6 (12.8%)**, Genotype-4 **n = 5 (10.6%)**, Genotype-5 **n = 2 (4.3%)**, Genotype-6 **n = 1 (2.1%)**; **Total N = 47**.							

Allele lengths are in base pairs (bp). A/B denote diploid allele copies per locus. Genotype labels are derived from multilocus profiles. Primer sequences (5′→3′) and fluorophore labels for CP1, CP4 and B5 microsatellite loci (as per Sabino et al., 2010)
