# Supplementary figures and images for: Silent Persistence: Molecular Evidence of Clonal Transmission in Fluconazole-Resistant Candida parapsilosis Hospital Outbreaks over Decades
Source: J Fungi (Basel). 2025 Nov 12;11(11):802. doi: 10.3390/jof11110802 (PMC12653143; doi:10.3390/jof11110802)

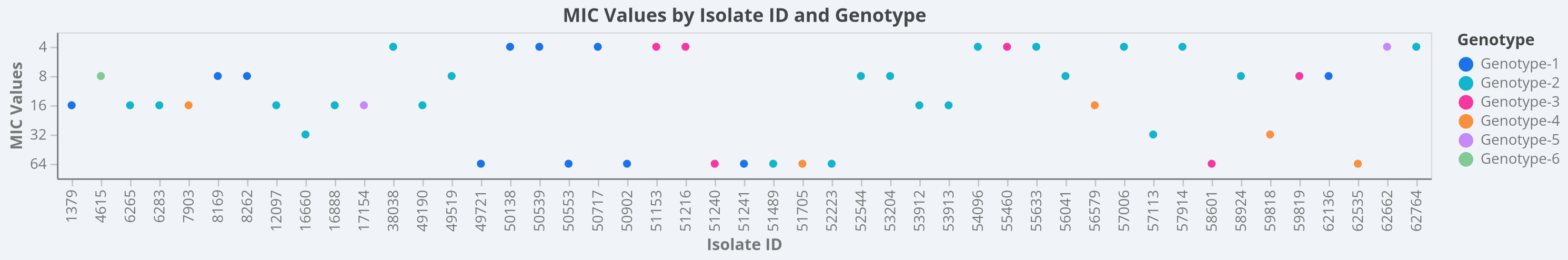

Supplement: Supplementary file 1 [file jof-11-00802-s001.zip › Supplementary Figure S1.png]

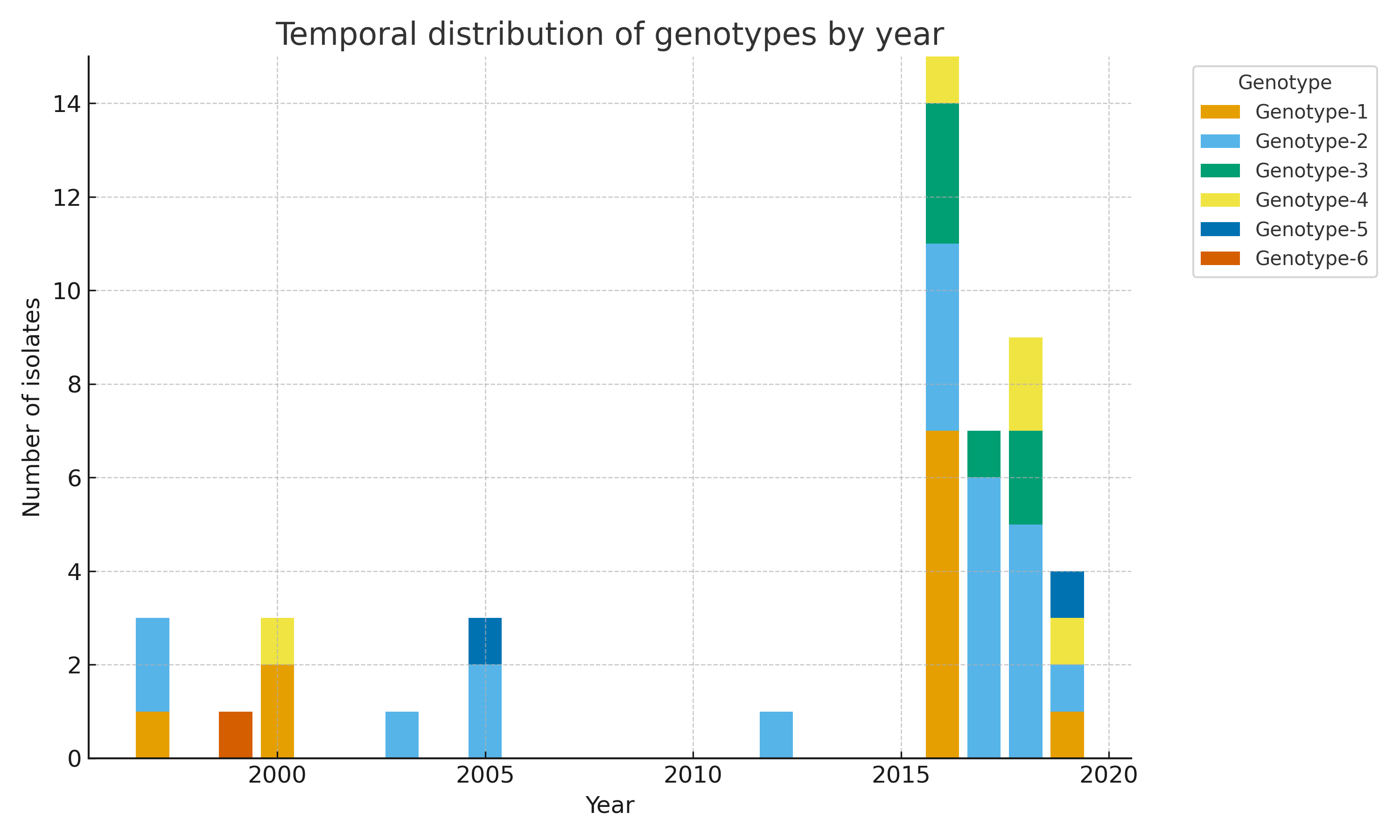

Supplement: Supplementary file 1 [file jof-11-00802-s001.zip › Supplementary Figure S2.png]
